# Tongue Acupuncture for the Treatment of Poststroke Aphasia: A Systematic Review and Meta-Analysis

**DOI:** 10.1155/2022/4731074

**Published:** 2022-10-03

**Authors:** Shengping Yang, Li Li, Rong Jiang, Haoying Ding, Fei Xu, Lulu Ge, Peng Kuang, Zuhong Wang

**Affiliations:** Kunming Municipal Hospital of Traditional Chinese Medicine, Kunming, Yunnan, China

## Abstract

**Objective:**

This review evaluated the efficacy of tongue acupuncture for the clinical treatment of poststroke aphasia.

**Methods:**

PubMed, Medline, Cochrane, Embase, CNKI, VIP, and Wanfang databases were searched from their inception to 1st June 2022. The dataset included randomized controlled trials (RCTs) with tongue acupuncture for the treatment of poststroke aphasia. Data aggregation and risk of bias evaluation were conducted on Review Manager Version 5.4.1 and Stata16.0. The main outcome measures included the Aphasia Battery of Chinese (ABC), the Chinese Functional Communication Profile (CFCP), the Boston Diagnostic Aphasia Examination (BDAE), and clinical efficiency. Then, comparing the effectiveness of tongue acupuncture, tongue acupuncture combined with conventional therapies, conventional therapies with head acupuncture, language training, body acupuncture, and Jie Yu Dan.

**Results:**

A total of 20 studies with 1355 patients were included. Meta-analysis showed that compared with conventional treatments, tongue acupuncture has a significant improvement in clinical efficacy score (MD = 1.25, 95% CI (1.09, 1.43), *P*=0.001) and CFCP of poststroke aphasia (MD = 39.78, 95% CI (26.59, 52.97), *P* < 0.00001), but was not significant in improving ABC (MD = 5.95, 95% CI (2.85, 9.04), *P*=0.06). Compared to the conventional treatments, tongue acupuncture combined with conventional therapies promoted the ABC (MD = 11.48, 95% CI (2.20, 20.75), *P* < 0.00001), clinical efficacy score (MD = 1.22, 95% CI (1.14, 1.30), *P* < 0.00001), and CFCP score (MD = 29.80, 95% CI (19.08, 40.52), *P* < 0.00001) of poststroke aphasia.

**Conclusion:**

This systematic review indicated that tongue acupuncture or tongue acupuncture combined with conventional treatments was an effective therapy for treating poststroke aphasia. However, stricter evaluation standards and rigorously designed RCTs are needed.

## 1. Background

Stroke is one of the most common neurovascular diseases, with a global prevalence of 101.5 million (2021, latest statistics on heart disease and stroke), among which ischemic stroke accounts for 76 percent (77.2 million) [1]. Ischemic stroke refers to ischemic necrosis or softening of localized cerebral tissue caused by cerebral blood circulation disorder, ischemia, hypoxia, and corresponding symptoms of neurological dysfunction [2]. Ischemic stroke is one of the serious diseases with the highest morbidity and mortality in the world. Stroke is the second leading cause of death among the top ten causes of death in the world, released by the World Health Organization [3]. It is also one of the main causes of severe disability and cognitive impairment worldwide [4].

However, the only effective treatment in the acute phase of ischemic stroke is the application of thrombolytic drugs. Although the emerging endovascular thrombectomy has extended the time window to 24 hours [5], most survived patients still have severe neurological symptoms. Poststroke aphasia (PSA) is one of the most devastating symptoms among the various functional disabilities caused by stroke [6]. More than one-third of stroke survivors suffer from aphasia, in which 30–43% remain chronically affected [7].

The main treatment measures recommended in the “Guidelines for the Diagnosis and Treatment of Cerebral Infarction in China (2017)” include syndrome differentiation and treatment combined with acupuncture and moxibustion [8]; in these guidelines, acupuncture therapy was recommended as a clinical physical therapy method with the exact curative effect on central nervous system diseases [9]. In recent years, acupuncture has made significant progress in the treatment of PSA. There are hypoglossal nerve, glossopharyngeal nerve, trigeminal nerve, and facial nerve distributed in the tongue, as well as rich peripheral nerve; the stimulating tongue can enhance the excitability of the central nervous system, promote nerve reflex, regulate the cortex-thalamus-cortex, balance the specific conduction and nonspecific conduction of the body, rebuild the neural circuit of language activity, and promote the recovery of language [10]. Many studies have shown that when the tongue is stimulated, the quiescent state of the language function of the cerebral cortex can be activated; then, the local stimulation acts as a communication circuit, which forms conditioned reflexes and improves language ability.

In the theory of traditional Chinese medicine, stroke damages the brain and spinal cord in the blood vessels, and its occurrence and recovery involve the dynamic pathological changes in multiple links of “blood-vessel-heart-spirit.” The heart opens at the tongue, and the heart is the official of the monarch. The heart area of the tongue is mainly used to treat diseases related to the heart, blood vessels, nerves, and the mental system, such as the Juquan acupoint is in the spleen and the stomach area of the tongue. The spleen manages the transportation and transformation of blood and qi. Therefore, stimulating specific acupoints of the tongue may have positive curative effects through the dynamic pathological changes of “blood-pulse-heart-spirit.”

Tongue acupuncture and its combination therapies (head acupuncture, language training, body acupuncture, and Jie Yu Dan) are gaining more and more attention from researchers, and some clinical studies have also described the clinical application of tongue acupuncture in the treatment of poststroke aphasia, but their quality has not been systematically evaluated. In addition, there was no meta-analysis for tongue acupuncture of poststroke aphasia in the past 10 years.

This study aims to critically evaluate the efficacy and safety of tongue acupuncture in the treatment of poststroke aphasia to provide an evidence base for the clinical practice of acupuncture.

## 2. Methods

### 2.1. Data Aggregation Method

We aggregated all the data about tongue acupuncture treatment after stroke aphasia RCTs from Chinese databases Chinese journal full-text database (CNKI), China biomedical literature database (CBM), VIP database (VIP), Wanfang data, and English databases PubMed, Cochrane, and Embase. The data aggregation time is from the establishment of the database to the present. Search terms include “stroke,” “cerebrovascular accident,” “cerebrovascular apoplexy,” “brain vascular accident,” “cerebrovascular stroke,” “cerebral stroke,” “acute stroke,” “acute cerebrovascular accident,” “CVA (cerebra-vascular accident),” “aphasia,” “post-stroke aphasia,” “randomized controlled trial,” “random allocation,” “randomization,” and “RCT.” The subject heading was combined with a free word search. The database retrieval strategy on CNKI are as follows: (1) Stroke AND acupuncture AND aphasia; (2) Stroke AND needle AND aphasia; (3) Cerebral hemorrhage AND needle AND aphasia; (4) Stroke AND acupuncture AND aphasia (in Chinese).

### 2.2. Inclusion Criteria

The inclusion criteria were as follows:

1. Randomized controlled trials (RCTs) of tongue acupuncture in the treatment of poststroke aphasia published in China and abroad
2. Patients who meet the diagnosis of poststroke aphasia, with comparable baselines. The treatment group used tongue acupuncture or combined tongue acupuncture based on the intervention measures in the control group.
3. The main efficiency measurements are the ABC score and clinical efficacy; the secondary efficiency measurement is the CFCP score.
4. The clinical efficacy observations and the experimental research on patients with tongue acupuncture as the main treatment
5. Reasonable control group, control group intervention measures (body acupuncture, head acupuncture, language training, and Jie Yu Dan)
6. The acupuncture points and methods are clearly exposed in the text

### 2.3. Exclusion Criteria

The exclusion criteria were as follows:

1. Studies not in line with the diagnosis of motor aphasia after stroke
2. The clinical treatment method is not mainly based on tongue acupuncture
3. Animal experiments, literature review, and meta-analysis literature
4. For repeated publications or repeated selections, keep one of them

### 2.4. Data Extraction

We imported the obtained literature data into Note Express software and used its automatic review function combined with manual review to remove duplicate literature. We reviewed all abstracts and full texts to make the first selection and carried out data extraction on the selected papers (basic research information, research methods, intervention measures, treatments, performance indicators, and literature quality evaluation).

### 2.5. Evaluation of the Quality of Research Methodology

We used Cochrane Handbook 5.4.1 “Risk of Bias Assessment Tool” to evaluate the selected studies, including random assignment, hidden grouping, blinding, incomplete data, selective reporting, and other sources of bias.

### 2.6. Statistical Methods

Statistical analysis was performed by using RevMan5.4.1 software provided by the Cochrane Collaboration. Publication bias analysis and sensitivity analysis were performed by using Stata16.0. The heterogeneity among the studies was tested through *I*^2^ and *P* tests. If *P* > 0.1 and *I*^2^<50%, a fixed effect model was used; otherwise, a random effects model was used.

For dichotomous variables, the relative risk (RR) and 95% confidence interval (CI) were used to represent the efficacy analysis statistics; for continuous data, weighted mean difference (MD) and 95% confidence interval (CI) were used to express efficacy analysis statistics. Potential publication bias was analyzed by using an “inverted funnel” diagram, and bias in included trials was discussed. Funnel plot and Egger's test were used to analyze the publication bias of the primary outcome indicators.

## 3. Results

### 3.1. Search Results

**Figure 1:**
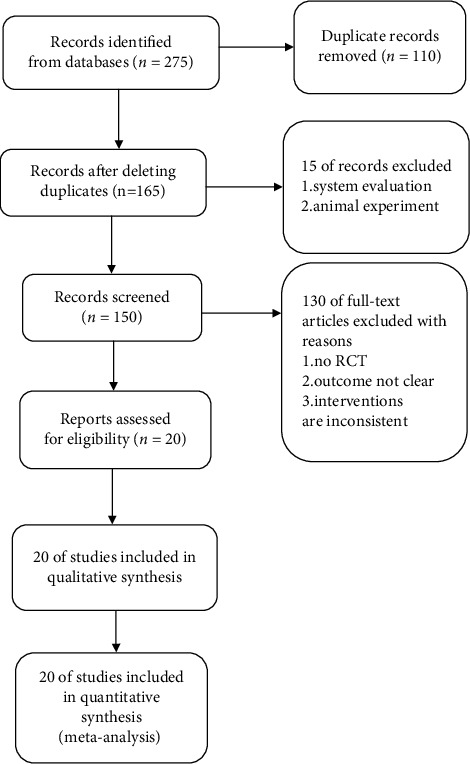
The flowchart of the screening process.

A total of 267 papers were found, among which 165 independent studies were deduplicated with the Note Express software. By browsing the abstract and reading the full text, they were screened according to the inclusion and exclusion criteria, and finally, 20 RCTs were included [11–30], with a total of 1355 patients, all studies having a comparable baseline. The literature screening process is shown in Figure 1. The basic characteristics of the included studies are listed in Table 1.

### 3.2. Quality of the Included Literature

**Figure 2:**
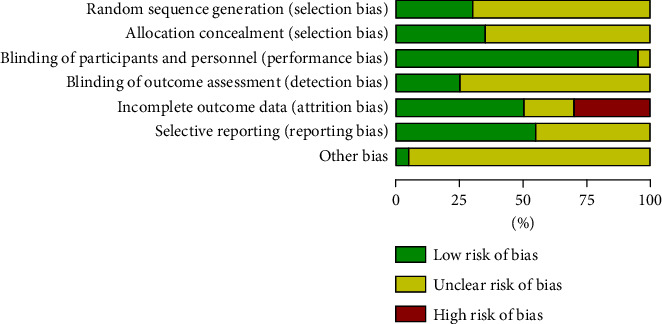
Risk of bias percentile bar graph.

**Figure 3:**
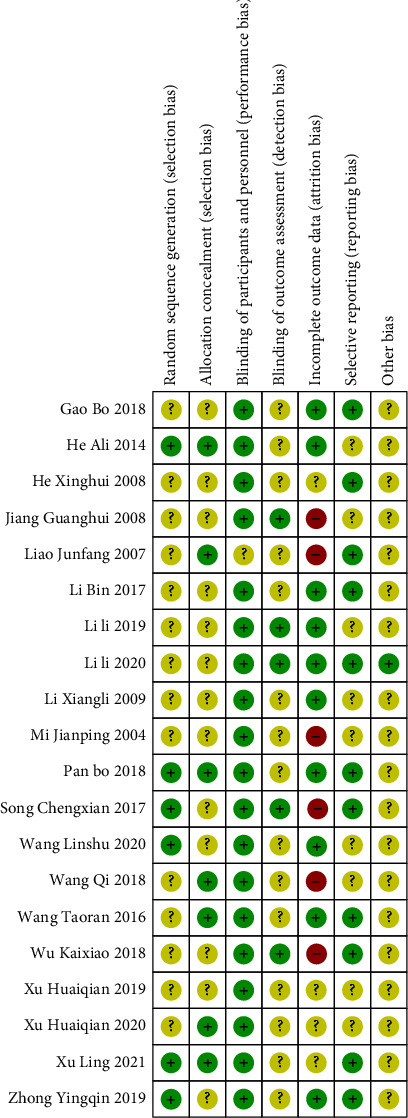
Risk of bias summary graph.

Cochrane risk of bias assessment was performed on the included literature, as shown in Figures 2 and 3. Three studies [13, 17, 21] used the random number table method, and the remaining studies [11, 12, 14–16, 18–20, 22–30] did not describe the specific randomization method. Four studies [19, 23, 29, 30] had no follow-up visits and dropouts. None of the studies have published reports, so it is impossible to judge whether the outcome of the choice to report exists. It is not clear whether other biases exist.

### 3.3. Meta-Analysis Results

#### 3.3.1. ABC Score

**Figure 4:**
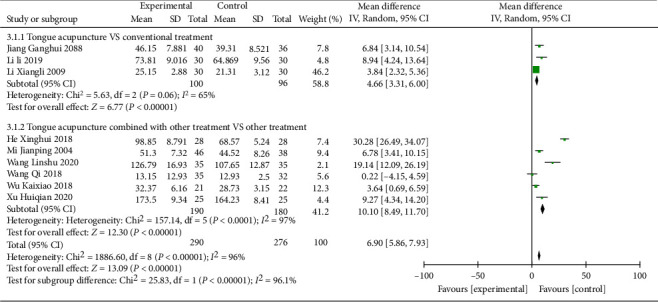
ABC score subgroup analysis.

Nine studies [13–15, 18, 19, 22, 27, 28, 30] reported ABC scores with large heterogeneity between studies (*P* < 0.00001, *I*^2^ = 96%). Subgroup analysis based on the intervention methods is shown in Figure 4.

**Figure 5:**
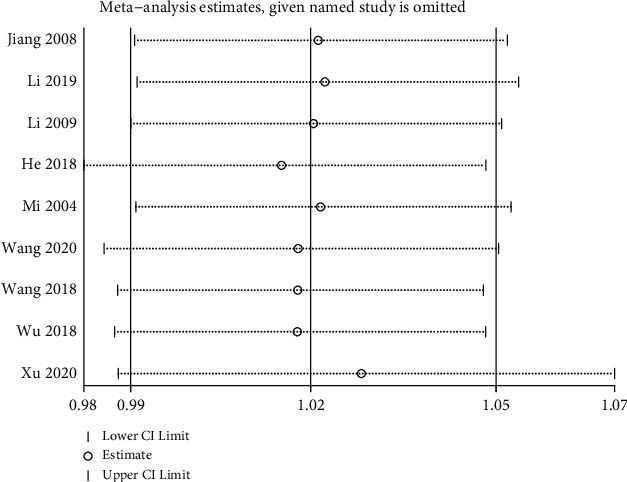
Sensitivity analysis ABC score.

The results of subgroup analysis showed that in the 3 studies comparing tongue acupuncture with conventional treatments [15, 27, 28] (MD = 5.95, 95% CI (2.85, 9.04), *P*=0.06), the difference between the two groups was not significant. Six studies comparing tongue acupuncture combined with conventional therapies and conventional therapies [13, 14, 18–20, 22] combined effect results (MD = 11.48, 95% CI (2.20, 20.75), *P* < 0.00001), indicating tongue acupuncture combined with conventional therapies increased the ABC score more compared with tongue acupuncture. Sensitivity analysis found no significant reversal of meta-analysis results, indicating that the results were robust. See Figure 5 for details.

#### 3.3.2. Clinical Efficacy

Fifteen studies [11–14, 16, 19–21, 23–27, 29, 30] reported clinical efficacy, with subgroup analyses by intervention methods. The results of subgroup analysis showed that the difference between tongue acupuncture and conventional treatments [12, 27, 29] was statistically significant (*P*=0.001).

**Figure 6:**
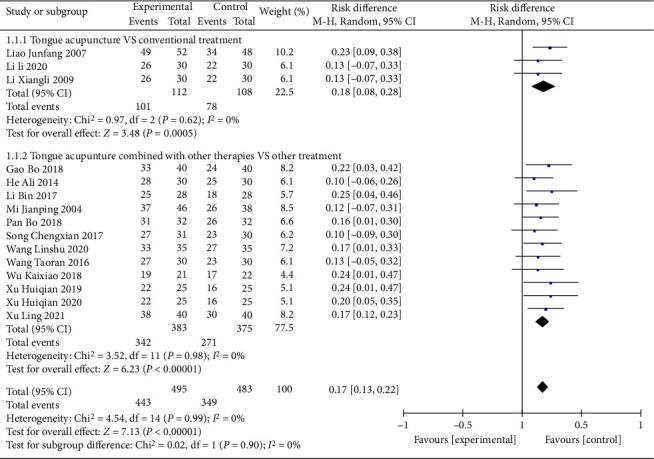
Clinical efficacy score of subgroup analysis.

**Figure 7:**
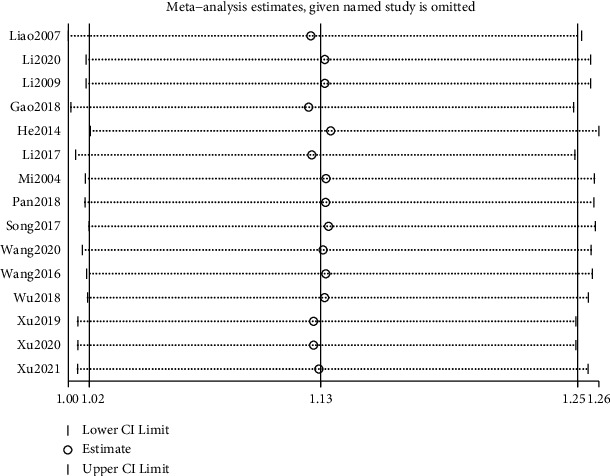
Sensitivity analysis of clinical efficacy.

The 12 studies that compared tongue acupuncture combined with conventional therapies to conventional therapies [11, 13, 14, 16, 19–21, 23–26, 30] showed good homogeneity (*P*=0.96, *I*^2^ = 0%), the difference was statistically significant (MD = 1.23, 95% CI (1.14,1.30), *P* < 0.00001), and the comparison of the two groups shows that tongue acupuncture combined with conventional therapies improved the clinical efficacy is clear. Details are shown in Figure 6. The results of sensitivity analysis showed that no studies had significantly reversed the results of meta-analysis, indicating that the above results were reliable. See Figure 7 for details.

#### 3.3.3. CFCP Score

**Figure 8:**
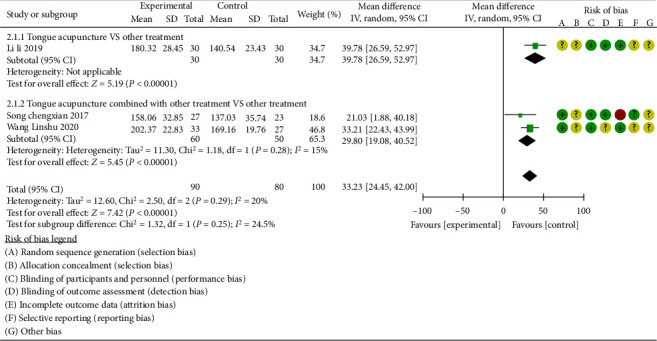
CFCP score of subgroup analysis.

Three studies [13, 15, 23] reported CFCP scores, and there was heterogeneity between studies (*P*=0.29, *I*^2^ = 20%), and subgroup analyses were performed according to different interventions (Figure 8). The results of subgroup analysis showed that the difference between tongue acupuncture and conventional treatments [15] was statistically significant (MD = 39.78, 95% CI (26.59, 52.97), *P* < 0.00001), indicating that tongue acupuncture can improve the CFCP score more compared with the conventional treatments.

There were two studies comparing tongue acupuncture combined with conventional therapies to conventional therapies [13, 23]. The difference between the studies was statistically significant (MD = 29.80, 95% CI (19.08, 40.52), *P* < 0.00001), indicating that tongue acupuncture combined with conventional therapies is more effective than conventional treatments in increasing the CFCP score.

### 3.4. Publication Bias Assessment

In this study, the funnel plot method was used to evaluate the publication bias of the primary outcome indicators, ABC score, and clinical efficacy.

#### 3.4.1. ABC Score Publication Bias

**Figure 9:**
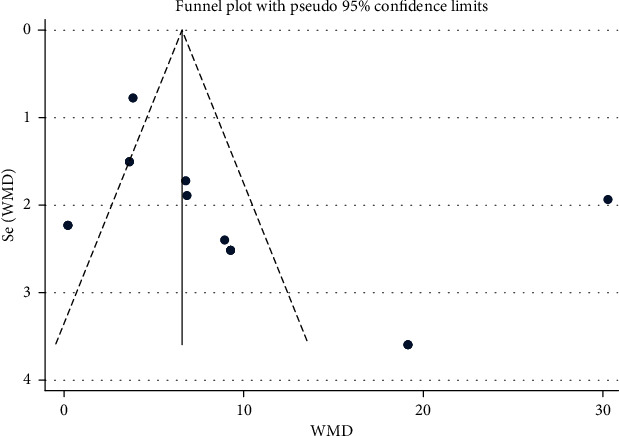
ABC score funnel chart.

Publication bias was evaluated for the 9 included studies [13–15, 18, 19, 22, 27, 28, 30]. The funnel plots show that two references comparing tongue acupuncture combined with conventional treatments to conventional treatments may present some publication bias (Figure 9. Egger's test was used for quantitative analysis; In Egger's test, *P* = 0.135 > 0.05, indicating no publication bias.

#### 3.4.2. Clinical Efficacy Publication Bias

**Figure 10:**
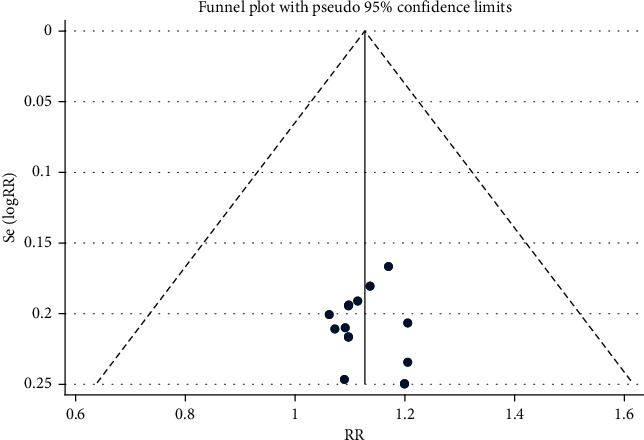
Clinical efficacy funnel chart.

The 15 included studies [11–14, 16, 19–21, 23–27, 29, 30] were evaluated for publication bias, and the funnel plot was roughly symmetrical, indicating that there may be no publication bias, as shown in Figure 10. Egger's test was used for quantitative analysis; In Egger's test, *P* = 0.201 > 0.05, indicating no publication bias.

## 4. Discussion

This systematic review confirmed the effectiveness of tongue acupuncture in the treatment of poststroke aphasia and indicated that tongue acupuncture combined with other therapies is effective in improving ABC scores, CFCP scores, and clinical efficacy.

Tongue acupuncture has a strong acupuncture sensation, and most of the clinical acupoints are mainly tongue acupoints, emphasizing the effect of near treatment. The tongue, as one of the main sound organs, can dredge the meridians and regulate qi and blood by stimulating the meridians and acupoints related to the tongue and sound. Tongue acupuncture is a microneedle therapy based on the theory of acupuncture and moxibustion in traditional Chinese medicine and modern bioholographic theory. It is a special acupuncture method created by Zhengzhou Guan, a famous acupuncture expert, based on the theory of the relationship between tongue and viscera meridians in Huangdi Neijing and his decades of clinical experience [31]. The 24 tongue acupoints relate to certain zang-fu organs. The distribution of tongue acupoints involves the relationship of mutual generation and constraining of five elements. It has become the basic acupoint of tongue acupuncture therapy. The commonly used tongue acupuncture includes the following steps: gargle with 3% hydrogen peroxide solution before acupuncture and ask the patient to extend the tongue naturally. By using disposable sterile acupuncture needles, twist the thumb evenly back and forth 10 times and insert the needles about 1–3 mm. For acupoints on the base of the tongue, the surgeon uses the left hand to fix the anterior 1/3 of the tongue with sterile gauze, so that the tongue is rolled up to expose the acupoints, and routine disinfection is performed. Then, use a long needle to puncture Jinjin and Yuye and choose 2 acupoints each time with bleeding.

In clinical application, tongue acupuncture can also be used in conjunction with other treatments, such as head acupuncture. Head acupuncture is a therapy to acupuncture the corresponding head area to treat diseases, which is based on the theory of zang-fu organs and meridians, combined with the functional localization principle of the cerebral cortex. The acupoints are generally emphasized less but more precisely. Head acupuncture can regulate the mind and restore consciousness to speed up the repair of the language function area of the cerebral cortex. Body acupuncture emphasizes holistic treatment and has the characteristics of combining syndrome differentiation with meridian differentiation and regulating the mind and qi. Puncture bloodletting therapy has the characteristics of the quick effect, remarkable curative effect, low cost, and few adverse reactions, but single bloodletting therapy is difficult to achieve long-term stimulation of the lingual nerve. Language training can re-establish the patient's language function through repeated stimulation from the aspects of oral movements, induced pronunciation, listening comprehension, and word writing. However, a single language training has a very long recovery period and needs to cooperate with clinical interventions to promote the recovery of patients, such as tongue acupuncture. Jie Yu Dan includes Qiang Huo and Quan Xie, which can relieve wind and dredge collaterals [21] and help the monarch medicine *Gastrodia elata* to dispel wind and phlegm; Quan Xie can also play an antispasmodic effect, which has a better prognosis for patients with cerebral infarction. Tongue acupuncture combined with Jie Yu Dan, the synergistic effect of the two, can enhance the efficacy of the drug to help patients improve speech function. Our results of meta-analysis confirmed that tongue acupuncture combined with the above therapies could make up for the limitations of each method alone, thus improving the clinical efficacy of aphasia patients.

The results of this meta-analysis showed that tongue acupuncture is beneficial to PSA. However, the underlying mechanism of tongue acupuncture action against PSA remains unclear. In recent years, many clinical studies and animal experiments have shown that acupuncture significantly improved clinical symptoms and the quality of life of patients by reducing the size of cerebral infarction, improving cerebral blood circulation, inhibiting apoptosis, and promoting cell proliferation and differentiation. Acupuncture therapy also has high safety with few adverse reactions and contraindications [32]. Some studies have suggested [33] that tongue acupuncture can reduce neuronal decay in the hippocampal CA1 region, rebuild cerebral nerve function, and play a positive role in restoring patients' language function. Tongue acupuncture can rapidly establish cerebrovascular collateral circulation [34], increase blood flow, improve cerebral circulation, and rebuild cerebral nerve activity after stroke by regulating the central nervous system. Studies have shown that [35] tongue acupuncture can significantly improve the blood perfusion of brain tissue at the lesion site more than body acupuncture and shrink the lesion site to varying degrees, thereby improving brain function. Tongue acupuncture can also reduce blood viscosity, improve microcirculation, prevent thrombosis, and enhance brain metabolism and blood supply to promote the repair of damaged brain tissue [19]. However, there is still a lack of research on the related signal pathways of tongue acupuncture in the treatment of poststroke aphasia, which should be the goal of future research.

A total of 20 studies were included in this study, and subgroup analysis was performed on CFCP scores [13, 15, 23] and ABC scores [13–15, 18, 19, 22, 27, 28, 30] according to intervention measures. The results showed that the difference between the tongue acupuncture and the conventional therapy group was statistically significant, but the heterogeneity was high, which may be related to the different acupoint selection and acupuncture intensity of the two groups, and less literature was included; the stability of the results is poor. In the ABC score publication bias, the funnel plots show that two references comparing tongue acupuncture combined with conventional treatments may present some publication bias, which may be due to the small number of cases included in the clinical control and cause shedding. More studies are needed to further analyze and verify the results. The analysis of tongue acupuncture combined with conventional therapy indicated that tongue acupuncture combined with conventional therapy for poststroke aphasia was superior to conventional therapy in improving CFCP score, ABC score, and clinical efficacy.

This review had certain limitations. The first limitation is the scarcity of studies, and the methodologically low to moderate quality of the primary data precludes us from drawing confirmative conclusions. Most of the included studies had an unclear risk of bias for blinding, random sequence generation, and allocation concealment; therefore, a preponderance of positive results was observed. The second limitation is that the number of studies is unevenly distributed in the different types of acupuncture, leading to a limited sample size for the CFCP score study. Meanwhile, the included studies also had limitations. The first limitation is that although mentioned randomization in all the 20 included studies, only 4 studies [12, 15, 16, 21] described specific randomization methods, and most of the included studies had unclear bias risks in blinding, random sequence generation, and allocation hiding. Although it is difficult for blind therapists who perform acupuncture, attempts should be made to blind patients, other care providers, and outcome evaluators to minimize trial outcome and evaluation bias. The second limitation is that the sample size included in the study was small, with the largest 120 cases [17] and the smallest 43 cases [19], and no sample size estimation was performed. A third limitation is that we did not perform a subgroup analysis of stroke duration, aphasia type, and treatment course, which may be a potential source of bias. These potential sources of clinical heterogeneity should be considered in future studies. This review also has some limitations. The first limitation is the scarcity of studies, and the methodologically low to moderate quality of the primary data precludes us from drawing confirmative conclusions. Most of the included studies had an unclear risk of bias for blinding, random sequence generation, and allocation concealment; therefore, a preponderance of positive results was observed. The second limitation is that the number of studies is unevenly distributed in the different types of acupuncture, leading to a limited sample size for the CFCP score study.

In the future, researchers should pay attention to the implementation of randomization, describe the randomization method in detail, improve the blind method and allocation concealment, standardize acupuncture therapy, focus on the records of adverse reactions and follow-up, try to select internationally recognized indicators, and improve the repeatability, quality, and reliability of research.

## 5. Conclusion

In conclusion, tongue acupuncture is effective and safe in the treatment of poststroke aphasia. Further exploration for tongue acupuncture of poststroke aphasia requires stricter evaluation criteria and high-quality RCT design.

## Figures and Tables

**Table 1:** Basic characteristics of the included literature.

Study	Patients number		Interventions		Treatment (days)	Results criteria
	Therapy group	Control group	Therapy group	Control group		
Xu et al. [11]	40	40	Tongue acupuncture + head acupuncture	Head acupuncture	60	⑤
Li et al. [12]	30	30	Jinjin, Yuye	Body acupuncture	20	⑥
Wang et al. [13]	35	35	Tongue acupuncture + head acupuncture + language training	Head acupuncture + language training	24	①②⑥
Xu et al. [14]	25	25	Tongue acupuncture + language training	Language training	40	②⑥
Li et al. [15]	30	30	Tongue acupuncture	Body acupuncture	20	①②③
Xu et al. [16]	25	25	Tongue acupuncture + language training	Language training	20	⑦
Zhong et al. [17]	60	60	Tongue acupuncture + head acupuncture + language training	Language training	30	②⑤
Wang er al. [18]	35	32	Tongue acupuncture + Schuell	Schuell stimulation	18	③
Wu et al. [19]	21	22	Tongue acupuncture + head acupuncture + language training	Language training	15	③④⑥
Gao et al. [20]	40	40	Tongue acupuncture + body acupuncture	Body acupuncture	15	④⑥
Pan et al. [21]	32	32	Tongue acupuncture + Jie Yu Dan	Jie Yu Dan	10	⑤
He et al. [22]	28	28	Tongue acupuncture + head acupuncture + Jie Yu Dan	Head acupuncture + Jie Yu Dan	24	②
Song et al. [23]	31	30	Tongue acupuncture + Schuell	Schuell stimulation	15	①③⑥
Li et al. [24]	28	28	Tongue acupuncture + language training	Language training	10	②⑥
Wang et al. [25]	30	30	Tongue acupuncture + language training	Language training	20	⑥
He et al. [26]	30	30	Tongue acupuncture + Schuell	Schuell stimulation	20	⑦
Li et al. [27]	30	30	Tongue acupuncture	Body acupuncture	10	③⑥
Jiang et al. [28]	40	36	Tongue acupuncture	Body acupuncture	20	②
Liao et al. [29]	52	48	Jinjin, Yuye	Body acupuncture	20	⑦
Mi et al. [30]	46	38	Tongue acupuncture + body acupuncture	Body acupuncture	30	②⑥

①, CFCP; ②, ABC; ③, BDAE; ④, NIHSS; ⑤, CADL; ⑥, clinical efficacy.

## Data Availability

The datasets analyzed during the current study are available from the corresponding author upon request.
